# Development of a high-resolution mass-spectrometry-based method and software for human leukocyte antigen typing

**DOI:** 10.3389/fimmu.2023.1188381

**Published:** 2023-04-28

**Authors:** Kun Wang, Zetao Sun, Fei Zhu, Yunping Xu, Feng Zhou

**Affiliations:** ^1^ Institutes of Biomedical Sciences, Fudan University, Shanghai, China; ^2^ Institute of Transfusion Medicine, Shenzhen Blood Center, Shenzhen, Guangdong, China; ^3^ Liver Cancer Institute, Zhongshan Hospital, Key Laboratory of Carcinogenesis and Cancer Invasion, Minister of Education, and Institutes of Biomedical Sciences, Fudan University, Shanghai, China

**Keywords:** HLA typing, mass spectrometry, nucleic acid mass spectrometry, cis/trans ambiguity, polymorphism

## Abstract

**Introduction:**

The human leukocyte antigen (HLA) system plays a critical role in the human immune system and is strongly associated with immune recognition and rejection in organ transplantation. HLA typing method has been extensively studied to increase the success rates of clinical organ transplantation. However, while polymerase chain reaction sequence-based typing (PCR-SBT) remains the gold standard, cis/trans ambiguity and nucleotide sequencing signal overlay during heterozygous typing present a problem. The high cost and low processing speed of Next Generation Sequencing (NGS) also render this approach inadequate for HLA typing.

**Methods and materials:**

To address these limitations of the current HLA typing methods, we developed a novel typing technology based on nucleic acid mass spectrometry (MS) of HLA. Our method takes advantage of the high-resolution mass analysis function of MS and HLAMSTTs (HLA MS Typing Tags, some short fragment PCR amplification target products) with precise primer combinations.

**Results:**

We correctly typed HLA by measuring the molecular weights of HLAMSTTs with single nucleotide polymorphisms (SNPs). In addition, we developed a supporting HLA MS typing software to design PCR primers, construct the MS database, and select the best-matching HLA typing results. With this new method, we typed 16 HLA-DQA1 samples, including 6 homozygotes and 10 heterozygotes. The MS typing results were validated by PCR-SBT.

**Discussion:**

The MS HLA typing method is rapid, efficient, accurate, and readily applicable to typing of homozygous and heterozygous samples.

## 1 Introduction

Human leukocyte antigen (HLA) plays a critical role in the human immune system. The HLA locus is located on the short arm of chromosome 6p21.3 and encodes cell-surface proteins. HLA molecules present self- or non-self-proteins to T cells, which orchestrates the adaptive immune response (1, 2). HLA genes in this region are known to be highly variable and form part of the most complex and polymorphic genetic system in humans. This variability is mainly because of single nucleotide polymorphisms (SNPs) within the HLA genes. Accurate HLA type identification is of great significance in organ and hematopoietic stem cell transplantation, where HLA matching is required for donors and recipients (3, 4). In the study of disease association, it is also important to accurately understand the individual HLA type because HLA allele variants have a strong genetic association with susceptibility to many human diseases, as well as drug sensitivity (5, 6). Littera et al. (7) discovered a protective effect of haplotype HLA-A*02:05, B*58:01, C*07:01, and DRB1*03:01 against SARS-CoV-2 infection. De Marco et al. (8) assessed the association between COVID-19 susceptibility and HLA polymorphisms in kidney transplant recipients. According to the research, the susceptibility to COVID-19 is associated with homozygosity at the HLA-A locus. Liu et al. (9) used single SNP analysis and Subset Testing and Analysis of Multiple Phenotypes (STAMP) to evaluate the association between HLA and many types of cancer. The study confirmed that infection-induced and hematopoietic malignancies show the strongest HLA correlations. The HLA locus is one of the most diverse and polymorphic regions in the human genome; more than 22,000 HLA alleles have been identified and reported by the IPD-IMGT/HLA database (IPD-IMGT/HLA release 3.47; Jan 2022) (10). To prevent alloreactivity, it is of crucial importance to reliably match the exact HLA allele type (11). For HLA typing, each HLA is assigned a unique name, which consist of four fields (an 8-digit number) of resolution separated by colons (i.e., HLA-A*01:01:01:01) (12).

In recent decades, DNA-based typing techniques have been used to identify HLA alleles, such as restriction fragment length polymorphism (RFLP), polymerase chain reaction-sequence specific oligonucleotides (PCR-SSO), PCR-sequence specific primers (PCR-SSP), and sequence-based typing (SBT) (13–15). However, RFLP, PCR-SSP, and PCR-SSO are low-resolution techniques with a high false positive or false negative rate, meaning they are often not suitable for reliable HLA identification at the allele level. While PCR-SBT remains the gold standard for HLA typing, cis/trans ambiguity in the heterozygous typing is a problem when two alleles are present and the composite sequence is identical for more than one combination (16, 17). Another issue that leads to inaccurate HLA typing is the difficulty in distinguishing between nucleotide and background signals in sequence overlays when allelic imbalance appears in heterozygous samples. With the development of Next-Generation Sequencing (NGS), some HLA laboratories have begun to use this method for HLA typing. However, NGS is expensive and the complex data generated is challenging to analyze and interpret. In addition, NGS of HLA is time-consuming, which is especially unfavorable in the context of deceased donor organ allocation (18–20). TA cloning and sequencing is an accurate method for resolving ambiguous Sanger sequencing results but it is labor- and time-intensive (21).

Mass spectrometry (MS) is an effective and rapid method for determining the molecular composition of a sample by measuring the mass-to-charge ratio (m/z) of its ions (22, 23). In MS, target molecules are ionized and become charged ions. Charged ions are accelerated by the electric field through the flight tube; the mass and charge of the ions affect their flight time. Therefore, the assayed compound is separated according to the time that each of its ions takes to reach the detector. Initially, this method predominantly found its application in the analysis of proteins and peptides because of its characteristics. Advances in nucleic acid charging and electrospray ionization methods have progressed the application of MS for nucleic acid and SNP analysis (24–26). In nucleic acid MS, the double-stranded DNA is dissociated into single strands, and the masses making up each strand are revealed in the mass spectrum. The detection of SNPs involves MS analysis in combination with multiplex PCR. Wandernoth et al. (27) developed a nucleic acid MS-based methodology and compared it with RT-PCR in many ways to rapidly detect SARS-CoV-2 from oral and nasopharyngeal swabs during the epidemic. Wu et al. (28) explored new SNP sites of miRNAs associated with gastric cancer by nucleic acid MS. Researchers classified miRNA SNP loci in 622 pairs of subjects and suggested rs7143252 could be used as a specific biomarker to screen for gastric cancer. Goyon et al. (29) analyzed single nucleotide impurities in therapeutic RNAs by MS. These impurities with the same length but slightly different polarity as their RNA parent molecules are located and quantified, providing insights for the further study of gRNA. Cao et al. (30) selected six SNPs to genotype 417 cases of IgA nephropathy and 424 healthy controls using MassARRAY MS and research indicated that rs17057846 and rs58747524 would increase the risk of IgA nephropathy in the Chinese Han population. Worrall et al. (31) first used MS as an alternative to hybridization-based typing methods, but it could not obtain high-resolution and accurate typing results to type HLA-DR. In addition, no definitive explanation could be given for the heterozygotes at this stage. The use of MS to type HLA, a gene region with the highest number of polymorphisms, is equally challenging and promising. Thus, this study aimed to develop a new MS-based high-resolution method for HLA typing, which has certain advantages in terms of cost and efficiency over existing methods. Our secondary goal was to solve the problem of ambiguity in the nucleotide sequence signal overlay generated using SBT.

To solve the limitations of SBT and the existing analysis software in terms of processing speed and result accuracy, we developed a method for rapid and accurate HLA typing and a supporting algorithm based on nucleic acid MS. The principle of MS-based HLA typing is that MS can detect small molecular weight changes in the analyte. The presence of SNPs in the HLA gene causes an obvious molecular weight change within the DNA fragment (e.g., when the base G is replaced by the base C, the molecular weight is reduced by 40 Da). The HLA MS typing algorithm and experimental method are based on changes in molecular weight and are capable of detecting HLA polymorphisms and enabling accurate HLA typing. Our newly established HLA typing method shows some cost and efficiency benefits. At the same time, due to the high-resolution characteristics of MS, heterozygote samples can be typed. In HLA MS typing, PCR plays a role in amplifying the SNP signal and making sure that samples meet the conditions for MS analysis, which is different from the role of PCR in PCR-SSP or PCR-SBT method. Therefore, MS-based HLA typing is a new typing method that does not rely on hybridization, fluorescence, or sequencing. In this study, we performed in silico experiment to test the performance of the HLA MS typing method and algorithm. We ensured that all validation samples were correctly typed according to the expected genotype. Ultimately, we used the HLA MS typing method to type a set of real HLA-DQA1 samples.

## 2 Materials and methods

### 2.1 HLA MS typing software

HLA MS typing software with self-written Python code was used to design PCR primers and construct the HLA MS database. A specific PCR primer pair was designed to amplify the HLA-DQA1 coding sequence (CDS), which spanned coding exons 1 to 4, using the GenBank database genomic references and the IPD-IMGT/HLA database (https://www.ebi.ac.uk/ipd/imgt/hla/). The HLA sequence data from the IPD-IMGT/HLA database were screened to eliminate alleles with frequencies lower than 0.5% in the Chinese population and to merge alleles with the same first 6-digit typing but different 7- and 8-digit typing IDs (32, 33).

A precise primer combination containing 30 pairs of primers was designed to amplify 30 different HLAMSTTs (HLA MS Typing Tags, some short fragment PCR amplification target products) according to the characteristics of the HLA-DQA1 conserved and variable regions. The primers were synthesized by Shanghai Sangon Biotech. The HLA-DQA1 MS database, holding data for 70 alleles, was constructed using the HLA MS typing software. The HLA-DQA1 MS database contains predicted molecular weight data, which will be searched and compared against the experimental molecular weight data.

### 2.2 cDNA acquisition

Blood samples were collected from the Shenzhen Blood Center, China. All subjects provided informed consent to participate in the study. All mRNA was isolated from peripheral blood mononuclear cells (PBMCs) using Maxwell^®^ RSC simplyRNA Blood Kit (Promega, USA) using the automated RNA preparation instrument, Maxwell^®^ CSC Instrument (Promega, USA). RNA purity was assessed by NanoDrop2000 (Thermo Fisher Scientific, USA), ensuring an optical density (OD) 260/280 ratio in the 1.9–2.0 range. The HiScript III 1st Strand cDNA Synthesis Kit (Vazyme, China) was used to obtain cDNA by reverse transcription according to the manufacturer’s protocol.

### 2.3 PCR amplification

The PCR was performed in two steps. In the first step, HLA-DQA1 was amplified in a 50 μL reaction volume consisting of 100 ng cDNA template (4 μL), 0.6 μM HLA-DQA1 primers (3 μL), and 2U TransStart TopTaq DNA Polymerase (0.8 μL, Transgen Biotech, China) with 10x Buffer (5 μL), 2.5 μM dNTPs (6 μL). The amplification reaction was performed on a Mastercycler Gradient (Eppendorf, Germany) using the following PCR cycling conditions: primary denaturation at 95°C for 3 min, followed by 30 cycles of 95°C for 15 s, 60°C for 1 min, 72°C for 30 s, and a final extension step at 72°C for 1 min. In the second step, 30 different HLAMSTTs were amplified with the 30 primer pairs designed using the HLA MS typing software. The amplification system and conditions were the same as for the first step of the PCR, except that the cDNA template was replaced with DQA1. We adopted strict negative controls for both the first and second PCR steps, which helped prevent possible contamination.

### 2.4 Purification and desalination of DNA

Briefly, 10 mg of Proteomix WAX (Sepax, China) was added to 30 different HLAMSTTs. The mixture was fully mixed and incubated at 37°C for 10 min. Next, the mixture was washed sequentially with 100 mmol/L sodium acetate (500 μL), then 40 mmol/L ammonium bicarbonate (500 μL), and finally 20% methanol (500 μL). Eventually, the purified HLAMSTTs were eluted with ammonia and dried by evaporation.

### 2.5 MS analysis

Purified HLAMSTTs were combined with 150 μL 50% acetonitrile and loaded onto TripleTOF 5600 (SCIEX, USA) to obtain mass spectra according to the following parameters: negative ion mode, ionspray voltage floating of 4500.0 V, temperature of 250.0°C, curtain gas of 20 L/h, ion source gas (Gas 1) of 25 L/h, and ion source gas (Gas 2) of 25 L/h.

### 2.6 Data analysis

The mass spectra were deconvoluted to calculate the molecular weight of HLAMSTTs using PeakView software (SCIEX, USA). The following parameters were used in the deconvolution: limited input m/z range from 600 to 1000; output mass range from 10000 Da to 30000 Da; step mass 1.0 Da; input spectrum isotope resolved resolution (30000); and H^+^ charge agent. The molecular weights were entered into the HLA MS typing software and the HLA typing results were generated by searching for corresponding molecular weights in the HLA-DQA1 MS database.

### 2.7 SBT typing

The reliability of the newly established MS typing method was validated by Sanger sequencing HLA-DQA1. PCR amplification of HLA-DQA1 exons 1 to 4 was performed for 16 samples with primers HLA-DQA1-F, 5’CAGAACAGCAACTGCTGAGG, and HLA-DQA1-R, 5’GGATGGGATTCACAATGGCC. The HLA-DQA1 PCR cycling parameters were set up as previously described (see section 2.3 on PCR amplification). The PCR products were separated by electrophoresis on a 1.2% agarose gel. The sequencing of clean DNA segments was performed by Shanghai Sangon Biotech (China).

## 3 Results

### 3.1 Experimental framework

**Figure 1:**
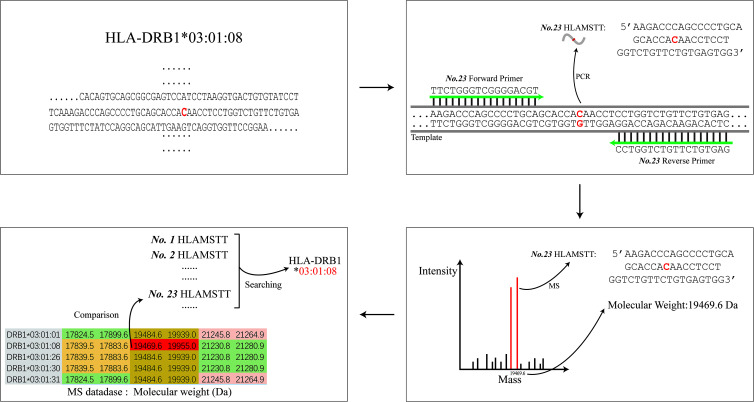
The schematic diagram of HLA MS typing. HLA MS typing software was used to perform SNP analysis of an HLA sample sequence and design PCR primers. Numerous PCR products containing SNPs were amplified and their molecular weights were obtained by MS analysis. The molecular weights from the experiments were used to interrogate the HLA MS database, and the typing result was eventually generated according to the search typing score.

In order to achieve the high-resolution HLA typing, primers designed by the HLA MS typing software amplified the DNA region with SNPs to generate short fragment PCR target products to differentiate an allele from others. These short fragment PCR target products with SNP information play an important role in HLA typing, which we call HLA MS Typing Tags (HLAMSTTs) in this article. For example, the allele HLA-DRB1*03:01:08 featured one SNP (rs77689370), differentiating it from other alleles (**Figure 1**). Only NO.23 HLAMSTT is shown in **Figure 1**. In reality, multiple pairs of primers were designed to amplify different HLAMSTTs, covering all SNPs with typing value. The molecular weight of each HLAMSTT was measured by MS and was used to interrogate the HLA MS database. Due to the large number of alleles at each HLA locus, it is necessary to use HLA typing software to establish an HLA MS database. The database contains amplification results and molecular weight data for each HLA allele, so that allele names correspond to the molecular weights of HLAMSTTs. Samples can then be typed according to the molecular weights obtained from the MS analysis. Therefore, when typing an unknown HLA gene, the molecular weights of multiple PCR products containing SNPs with typing value can be amplified by PCR, and accurate 6-digit HLA typing results can be obtained by searching the HLA MS database.

### 3.2 HLA MS typing algorithm and software

**Figure 2:**
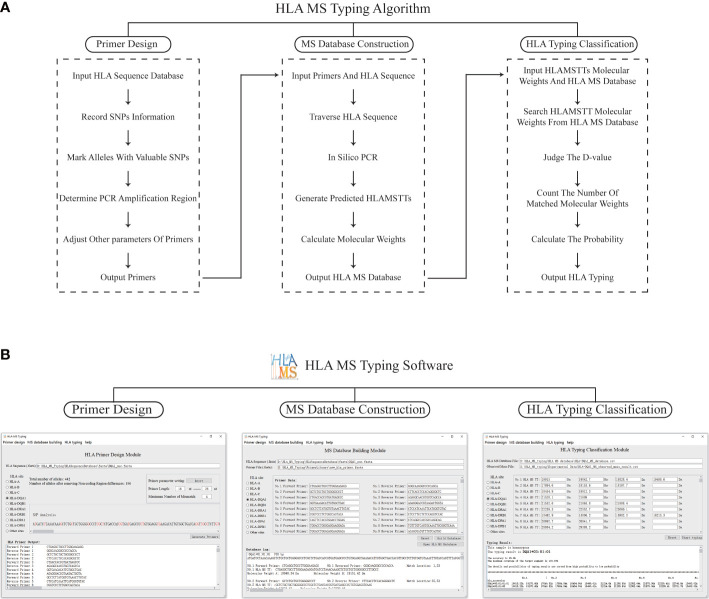
The schematic diagram and software interface of the HLA MS typing algorithm. **(A)** The HLA MS typing algorithm includes three modules: the primer design module, the database construction module, and the HLA typing classification module, which respectively complete the functions of PCR primer design, constructing the HLA MS database, and generating the HLA typing results following interrogation of the HLA MS database. **(B)** Three modules of the user interface: it mainly implements the import of HLA sequences, the export and import of PCR primers, the export and import of the HLA MS database, the output of typing results, typing scores, and detailed alignment results.

The HLA MS typing software is a visual MS data processing tool based on Python 3.7. The software has three modules: PCR primer design, HLA MS database construction, and HLA typing classification (**Figure 2A**).

In the PCR primer design module, each base of an HLA sequence was retrieved after alignment to determine the presence of an SNP and record the SNP variant information. For example, group 1 (which includes DRB1*01, DRB1*15, DRB1*16 and DRB1*12:17) was base A at the 14th base in exon 1 and group 2 (which includes other HLA alleles) was base G at the same base position. All alleles were annotated with SNP information to distinguish one allele from all other alleles by one SNP or a group of SNPs, while keeping the number of SNPs in the SNP group to a minimum. These SNPs, which were used to annotate alleles, were referred to as “SNPs with typing value”. PCR primers were designed according to the location of SNPs with typing value on the genome. First, forward and reverse primers were located on two flanks of the SNPs with typing value to ensure a valid amplification of HLAMSTTs. A maximum of three primer-template mismatches were tolerated (especially at the 3’ terminus of the primer) to improve amplification efficiency. We ensured that the length difference between the full set of primers did not exceed 8 bp, while the annealing temperature difference for all primers did not exceed 7 °C. The above parameters could be adjusted within the HLA MS software. Finally, the software verified the validity and specificity of the primers: match the primers with all HLA sequences respectively, judge the amplification efficiency according to the number of mismatch bases, and determine whether the SNPs in HLAMSTTs meet expectations. The PCR primers were used to amplify the HLA gene region containing SNPs into HLAMSTTs with a length of 50–100 bp to meet the requirements of MS for nucleic acid analysis and amplify MS signal.

The HLA MS database construction module predicted the expected HLAMSTT and molecular weight of each allele by in silico PCR. In this module, in silico PCR was implemented in Python using the Python library FuzzyWuzzy. The PCR template was divided into different fragments with the same length as the primer. These fragments were aligned with the primer separately, using the Python function “process.extractone”. The fragment most similar to the primer sequence was selected, and the location of this fragment was the primer binding site on the template. The in silico HLAMSTT comprised a forward primer sequence, an extended sequence in which SNPs were present, and a reverse primer sequence. We calculated the molecular weight of HLAMSTT, according to the molecular weight and number of each deoxyribonucleotide. Two single strands of DNA were calculated separately, because the double strands of DNA would be untied in MS. The procedure was repeated for each allele to construct the HLA MS database, which linked HLA type to a particular primer pair and molecular weight.

In the HLA typing classification module, the software was used to compare the molecular weights of HLAMSTTs measured by MS with the those in the HLA MS database. A match between the molecular weights generated the HLA typing result. When the molecular weight difference (between the HLAMSTT measured by MS and that in the HLA MS database) was equal to or less than ±1 Da, the HLAMSTT was considered close enough to map a known allele in the database. When multiple molecular weights of HLAMSTTs from an unknown sample were input, the ‘typing score’ of each allele in the database was calculated as the ratio between: 1) the number of molecular weights mapped to the HLAMSTTs; and 2) the total number of molecular weights. For each allele, the higher the number of molecular weights mapped to HLAMSTTs from an unknown sample, the higher the typing score is, and thus, making it more likely to identify that unknown sample as the allele.

**Figure 2B** illustrates how HLA MS typing software functions. In the PCR primer design module, the user selects HLA sites from the software user interface, imports the FASTA file of HLA sequence data from the IMGT/HLA database, and sets primer parameters. Next, the results of SNP analysis and the best primer combination are shown in software. Using this function, we designed primers for HLA-DPA1, -DPB1, -DQA1, -DQB1, -DRA, and -DRB. In the HLA MS database construction module of the software, after selecting the HLA site, the user imports the HLA sequence FASTA file, followed by the primer sequence FASTA file. Alternatively, the user can manually input the primer sequence. The software then generates the output in the form of an HLA MS database CSV file. Using this function, we established the HLA MS database for six HLA loci: HLA-DPA1, -DPB1, -DQA1, -DQB1, -DRA, and -DRB. To use the HLA typing classification module, the user imports the MS database CSV file and the CSV file of molecular weight data measured by MS. Alternatively, the user can manually input the sample molecular weight data measured by MS and set the threshold value (Default is 1 Da) to determine whether there is a match between molecular weights. The software output includes the HLA typing result, the associated heterozygosity, the typing score, and a detailed comparison. If the tested sample is heterozygous, the software displays at least two high confidence alleles and provides heterozygous typing information.

### 3.3 Practical application of HLA-DQA1 typing

**Figure 3:**
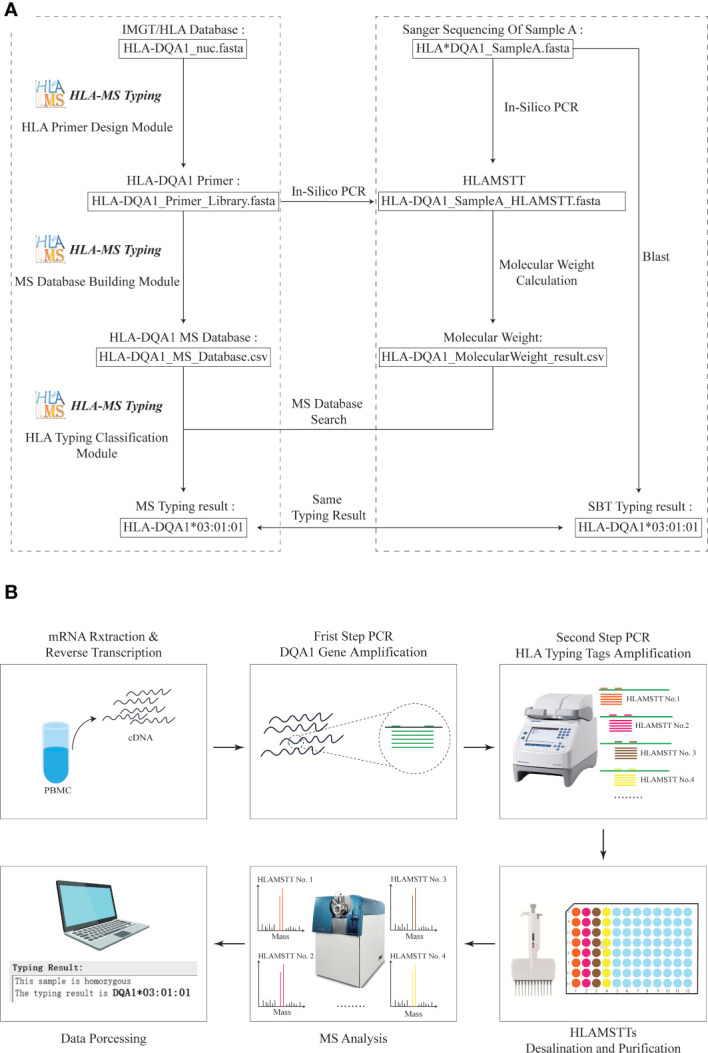
Practical application of HLA MS typing in silico and wet validation. **(A)** HLA MS typing in silico validation with HLA-DQA1. For HLA samples with known Sanger sequencing results, SBT and HLA MS typing were used simultaneously for in silico typing, and the same typing results were obtained; HLA-DQA1*03:01:01. **(B)** HLA-DQA1 MS typing using wet laboratory techniques. For an untyped HLA-DQA1 sample, the following process was performed: mRNA extraction and reverse transcription, DQA1 gene amplification, HLAMSTT amplification, MS typing, and data processing; the typing result was HLA-DQA1*03:01:01.

To ensure the validity of the HLA MS typing algorithm and software, we first used HLA-DQA1 test samples with known sequences for in silico verification (**Figure 3A**). The workflow and final typing results generated by the HLA MS typing software are presented in the left panel of the dotted box. The PCR primer design module generated primer combinations based on the IMGT/HLA database. The MS database construction module converted the primer data into PCR products and molecular weights. The HLA typing classification module assigned the HLA-DQA1*03:01:01 typing result by matching the molecular weights of the test sample to those in the HLA MS database. The right panel of the dotted box showed the workflow and the test sample typing result based on Sanger sequencing. HLA-DQA1 Sanger sequencing and primer data were subjected to in silico PCR. We also entered the Sanger sequencing data into the Basic Local Alignment Search Tool (BLAST), and obtained the HLA-DQA1*03:01:01 typing result. The typing results obtained by the two methods were the same, which confirmed the feasibility of MS method in silico level.

Sixteen blood samples were used to verify the validity of the HLA MS typing method and algorithm in a wet laboratory experiment (**Figure 3B**). This involved mRNA extraction and reverse transcription (3 h), DQA1 gene amplification (2 h), HLAMSTTs amplification (2 h), desalination and purification (1 h), MS analysis (2 h), and data processing (1 h). A parallel experimental workflow was performed, which vastly improved the speed of sample analysis theoretically. The total experimental time is less than 12 hours for 16 samples.

**Figure 4:**
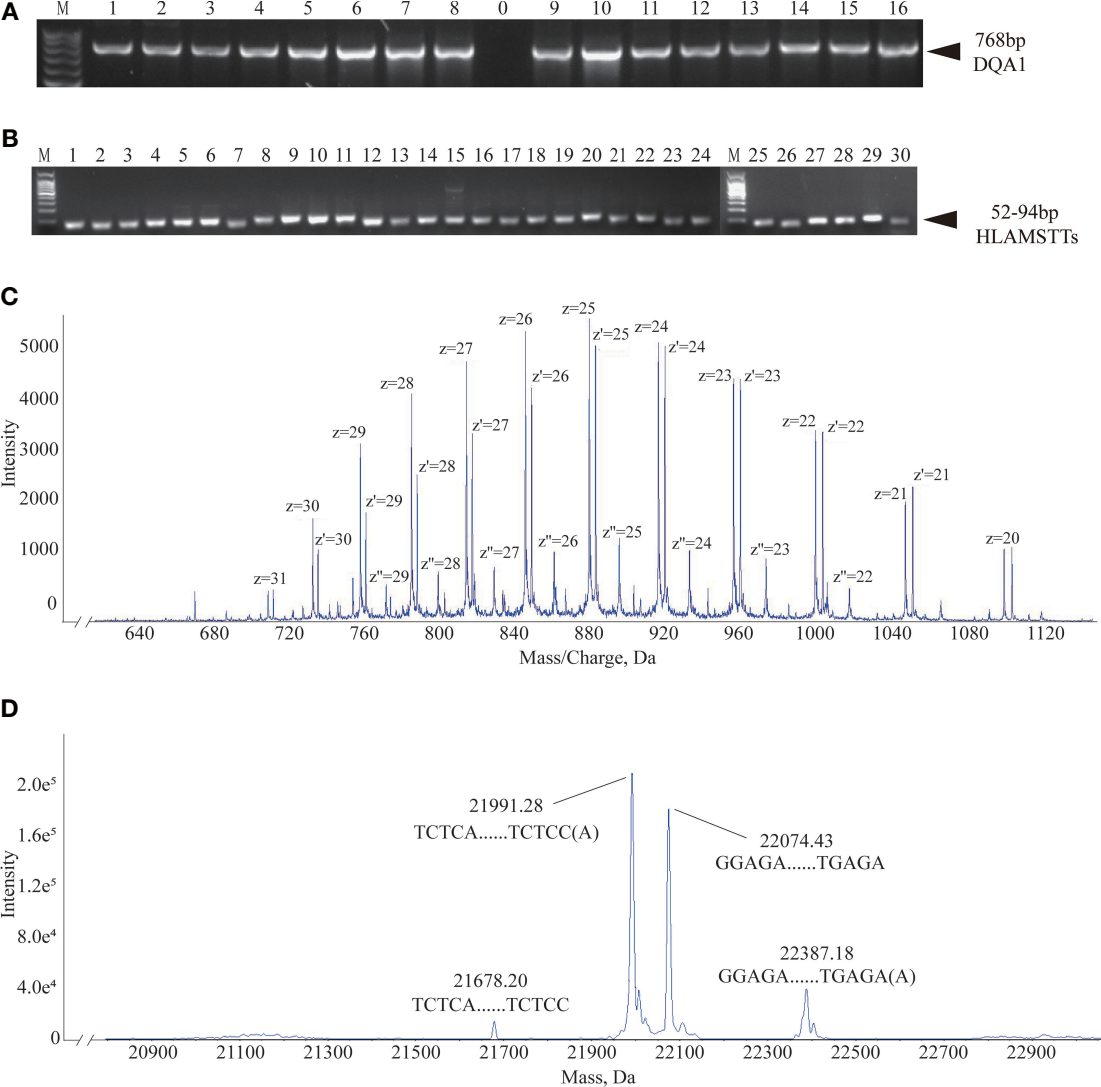
Agarose gel electrophoresis and MS data interpretation. **(A)** Agarose gel electrophoresis of 16 HLA-DQA1 showed bands at 768 bp. **(B)** HLAMSTT gel electrophoresis of Sample 2 showed bands at 52–94 bp. **(C)** Mass spectrum of Sample 2 No. 26 HLAMSTT with corresponding mass-to-charge ratio (m/z) peaks. The m/z of the HLAMSTT was between 700 and 1100 Da, and the maximum intensity of the signal was between 5000 and 6000. **(D)** Mass spectrum of Sample 2 No. 26 HLAMSTT with corresponding molecular weight peaks. The molecular weights of the two highest peaks were 21992.11 Da and 22075.32 Da, corresponding to the DNA single strands TCTCAGGCTCCCAGTGTTTCAGAAGAGGCTGGTCCAGGCCCCAGTGCTCCACCTTGCAGTCATAACTCTCC(A) and GGAGAGTTATGACTGCAAGGTGGAGCACTGGGGCCTGGACCAGCCTCTTCTGAAACACTGGGAGCCTGAGA, respectively; their intensities were 1.8e5 and 1.5e5, respectively.

Based on the primer combinations predicted by the HLA MS typing software (**Table 1**), optimal PCR amplification conditions and systems were determined, including annealing temperature and time, extension time, ratio of primers and templates, and concentration of dNTPs and Mg2^+^. Stable and specific amplification of HLA-DQA1 gene products by PCR was conducted for all samples; clear bands were visualized on an agarose gel at 768 bp (**Figure 4A**). Specific primer combinations stably amplified polymorphic regions, generating 30 different HLAMSTTs, carrying all SNPs with typing value. The length of HLAMSTTs ranged from 52 to 94 bp and the bands were visualized on an agarose gel (**Figure 4B**).

**Table 1:** Specific primer combinations.

Primer name	Forward primer	Reverse primer
DQA1 primer	CAGAACAGCAACTGCTGAGG	GGATGGGATTCACAATGGCC
Primer 1	CTGAGGCTGCCTTGGGAAGAGG	GGGCAAGGGCCCCCAGCA
Primer 2	GCTCTGCTGCTGGGGGCCCT	CTTCACCTCCACAGGGGCTC
Primer 3	CTGACCACCGTGATGAGCCC	AGAGGCAACGTGGTCAGCCA
Primer 4	GGTGAAGACATTGTGGCTGAC	AGAGGGACCGTAAGACTGGTA
Primer 5	GCCTCTTATGGTGTAAACTTGTAC	CTCCATCAAATTCATGGGTGTAC
Primer 6	CGGTCCCTCTGGCCAGTACA	CTCCTTCCTCTCCAGGTCCAC
Primer 7	CAGTTCTACGTGGACCTGGAG	CTCAGGCCACCGCCAGGCAG
Primer 8	TGGACCTGGGGAGGAAGGAGA	TGTTTGTCAGTGCAAATTGCGGGTCAAA
Primer 9	TGGACCTGGAGAGGAAGGAGA	ACAGCGATGTTTGTCAGTGC
Primer 10	ACCTGGGGAGGAAGGAGACTG	ACAGCCATGTTTGTCAGTGCA
Primer 11	ACCTGGGGAGGAAGGAGACTG	ACAGCCATGTTTGTCA
Primer 12	TGCACTGACAAACATGGCTGT	AGAGTTGGAGCGTTTAATCA
Primer 13	TGCACTGACAAACAT	AGAGTTGGAGCGTTTAATCA
Primer 14	TGCACTGACAAACATCGCTGT	GCGGTAGAGTTGGAGCGTTTA
Primer 15	CACAACTTGAACATCGTGATTAA	CAGGAACCTCATTGGTAGCAG
Primer 16	TAAACGCTCCAACTCTACCGC	TTGGAAAACACTGTGACCTCA
Primer 17	CTGCTACCAATGAGGTTCCTGAG	AGTGTCACGGGAGACTTGGAA
Primer 18	CACAGTGTTTTCCAAGTCTCC	ACAAGACAGATGAGGGTGTTG
Primer 19	GTGACACTGGGTCAGCCCAACA	CAGGAGGAAAGATGTTGTCCAC
Primer 20	GGACAACATCTTTCCTCCTGT	TCAGAAACACCTTCTGTGACTG
Primer 21	ATGGGCACTCAGTCACAGAAG	ATGATCACTCTTGGAGAGGAAG
Primer 22	ACCAGCTTCCTCTCCAAGAGTG	AGAAGGGAGGAAGGTGAGG
Primer 23	CCTTCTTCAAGATCAGTTACCTC	AATCTCATCAGCAGAAGGGA
Primer 24	ACCTCACCCTCCTCCCTTCT	TGCTCCACCTTGCAGTCATAA
Primer 25	GGAGAGTTATGACTGCAAGGTG	TCTCAGGCTCCCAGTGTTTCAG
Primer 26	AAACACTGGGAGCCTGAGATTC	ACCACAGTCTCTGTGAGCTCT
Primer 27	CCCCTATGTCAGAGCTCACAG	AATGCCCACGAGGCCCACAGA
Primer 28	CCCCTATGTCAGAGCTCACAG	ATGCCCACGAGGCCCACAGAC
Primer 29	GGGTTGTCTGTGGGCCTCGTG	GTGTCTGGAAGCACCAACTGAA
Primer 30	AGTTGGTGCTTCCAGACACCA	TACCTTCCCTTCCAGGATGGGAT

The desalted and purified HLAMSTTs were subjected to MS, and the two single strands of HLAMSTTs were separated under soft ionization. The two DNA single strands are shown on the spectrum in the form of m/z values (**Figure 4C**). The same DNA single strand could have different charges and therefore different m/z values. MS analysis showed that the m/z of HLAMSTTs was between 700 and 1100 Da, and the maximum intensity of the signal was between 5000 and 6000. The regular response signal indicated that the purification and desalination methods used for DNA preparation and nucleic acid MS were stable and effective. The m/z peaks were converted into four molecular weight peaks by deconvolution (**Figure 4D**). The molecular weights of the two highest peaks were 21992.11 Da and 22075.32 Da, corresponding to the DNA single strands TCTCAGGCTCCCAGTGTTTCAGAAGAGGCTGGTCCAGGCCCCAGTGCTCCACCTTGCAGTCATAACTCTCC(A) and GGAGAGTTATGACTGCAAGGTGGAGCACTGGGGCCTGGACCAGCCTCTTCTGAAACACTGGGAGCCTGAGA, respectively. The intensities of these peaks were 1.8e5 and 1.5e5, respectively. We also observed two further peaks with intensities lower than 6.0e4 on the spectrum, because a non-template AMP (313.21 Da) was added to the 3’ end of HLAMSTTs during PCR amplification (34). Therefore, an increase in molecular weight by ~313.2 Da (compared to the expected molecular weight) could be observed in mass spectrum signal (22387.68 − 22075.32 = 312.36; 21992.11 − 21679.02 = 313.09). The phenomenon could be considered as a two-fold validation for the recognition of molecular weights. We accurately typed 16 HLA-DQA1 samples (including 6 homozygotes and 10 heterozygotes) and obtained 6-digit typing results by MS-based typing method (**Table 2**). These HLA typing results were confirmed by SBT. However, for some low-quality and heterozygous samples, SBT method generated ambiguous typing results, in addition to being costly and labor-intensive. In contrast, HLA MS typing was less dependent on the quality of templates and was better at typing heterozygous HLA samples.

**Table 2:** HLA-DQA1 typing results.

Sample ID	HLA-DQA1 (1)	HLA-DQA1 (2)
1	06:01:01	01:01:01
2	01:02:01	01:02:02
3	01:03:01	03:02:01
4	03:01:01	03:01:01
5	03:03:01	03:03:01
6	01:03:01	03:01:01
7	03:03:01	06:01:01
8	01:03:01	01:02:02
9	03:02:01	03:02:01
10	02:01:01	02:01:01
11	02:01:01	02:01:01
12	03:02:01	03:02:01
13	01:04:01	01:05:01
14	06:01:01	06:01:01
15	03:01:01	06:01:01
16	03:03:01	05:05:01

### 3.4 Typing of heterozygous HLA samples

In heterozygous samples, the sequences of the two alleles are different and the molecular weights of HLAMSTTs with SNPs are different. We initially used in silico data to test the effectiveness of our HLA MS typing software in typing heterozygous samples. HLA-DQA1*01:03:01 and HLA-DQA1*01:01:01 were randomly selected from the HLA database to simulate two alleles in a heterozygous sample. In silico HLAMSTT and molecular weights of the two alleles were obtained using the HLA MS typing software. The molecular weights of No. 1, No. 2, No. 3, No. 5, Nos. 8–18, Nos. 21–26, and No. 29 HLAMSTTs of the two alleles were the same, and the molecular weights of No. 4, No. 6, No. 7, No. 19, No. 20, No. 27, No. 28, and No. 30 HLAMSTTs were different. The molecular weights were sequentially entered into the HLA typing classification module of the HLA MS typing software. The software judged that the molecular weight data originated from two different alleles, and that the samples were heterozygous. The software produced two typing results with the highest scores, namely, HLA-DQA1*01:03:01 and HLA-DQA1*01:01:01, which showed the ability of the HLA MS typing software to classify heterozygous samples.

**Figure 5:**
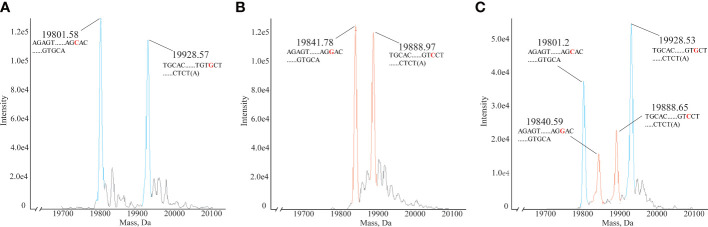
Mass spectra of homozygous and heterozygous samples. **(A)** Mass spectrum of homozygous Sample 4 No. 12. HLAMSTT, typing: HLA-DQA1*03:01:01, molecular weights: 19801.58 Da and 19928.57 Da. **(B)** Mass spectrum of homozygous Sample 10 No. 12. HLAMSTT typing: HLA-DQA1*02:01:01, molecular weights: 19841.78 Da and 19888.97 Da. **(C)** Mass spectrum of heterozygous Sample 16 No. 12. HLAMSTT typing: HLA-DQA1*03:03:01 and HLA-DQA1*05:05:01, molecular weights: 19801.2 Da, 19840.59 Da, 19888.65 Da, and 19928.53 Da.

In clinical HLA-DQA1 typing, heterozygous samples account for a large proportion. No. 12 HLAMSTTs was selected from three clinical samples, namely Sample 4 (**Figure 5A**), Sample 10 (**Figure 5B**), Sample 16 (**Figure 5C**), and analyzed using the HLA MS typing method. **Figure 5A** showed the molecular weights of the two DNA single strand peaks: 19801.58 Da and 19928.57 Da. According to the HLA MS database search, the standard molecular weights were 19800.96 Da and 19928.05 Da, which differed by only 0.62 Da and 0.52 Da from the experimental molecular weights measured by MS, respectively. The base sequences were: AGAGTTGGAGCGTTTAATCACGATGTTCAAGTTATGTTTTAGCACAGCCATGTTTGTCAGTGCA and TGCACTGACAAACATGGCTGTGCTAAAACATAACTTGAACATCGTGATTAAACGCTCCAACTCT(A). **Figure 5B** also showed the molecular weights of two DNA single strand peaks: 19841.78 Da and 19888.97 Da. According to the HLA MS database search, the standard molecular weights were 19840.99 Da and 19888.02 Da, which were only 0.79 Da and 0.95 Da away from the experimental molecular weights, respectively. The base sequences were: AGAGTTGGAGCGTTTAATCACGATGTTCAAGTTATGTTTTAGGACAGCCATGTTTGTCAGTGCA and TGCACTGACAAACATGGCTGTCCTAAAACATAACTTGAACATCGTGATTAAACGCTCCAACTCT(A). **Figure 5C** showed four molecular weight peaks: 19801.2 Da, 19840.59 Da, 19888.65 Da, and 19928.53 Da. According to the HLA MS database search, the standard molecular weights were 19800.96 Da, 19840.99 Da, 19888.02 Da, and 19928.05 Da, which differed by only 0.24 Da, 0.4 Da, 0.63 Da, and 0.53 Da, respectively, from those in the experimental molecular weights. We concluded that the sample contained sequences belonging to two different HLAMSTTs. The four peaks presented in **Figure 5C** corresponded to two different alleles in the HLA MS database, which confirmed the heterozygosity of this sample. Using No. 12 HLAMSTT and other HLAMSTTs MS data from the three samples, the HLA MS typing software typed the HLA as follows: Sample 4, homozygous, HLA-DQA1*03:01:01; Sample 10, homozygous, HLA-DQA1*02:01:01; and Sample 16, heterozygous, HLA-DQA1*03:03:01 and HLA-DQA1*05:05:01.

## 4 Discussion

HLA typing information is crucial to ensure correct HLA matching in solid organ transplantation. MS technology has become an important method for characterizing SNPs and genotyping. However, since there is no published literature proving the advantages of MS for the analysis of multi-target DNA fragments, no extensive studies have demonstrated the accuracy of MS typing in highly polymorphic gene regions, such as HLA genes. This study aims to develop a comprehensive MS typing method for characterizing HLA genes to improve existing HLA typing methods so that HLA polymorphisms can be more accurately determined for clinical use.

In this study, the algorithm and experimental workflow of HLA typing were based on the molecular weight of HLAMSTTs. We used the new typing method to type HLA-DQA1 and generate 16 high-resolution typing results, which demonstrated its high speed and accuracy. In MS HLA typing, the HLAMSTTs are amplified by a software-designed primer combination. Meanwhile, the molecular weights of purified HLAMSTTs are analyzed by MS. Experimental data are processed by the software and the HLA typing result is automatically generated. The frequency of DQA1 alleles included in the study accounts for 97.1% of all alleles found in the Chinese population, demonstrating the universality of MS HLA typing method. The parallel experimental workflow also has the advantage in terms of speed, which is especially advantageous in the typing of samples from deceased donors. Synthesized primers, DNA polymerase, Mg^2+^, dNTP, and other readily available reagents are mixed and added into a 96-well PCR plate, prior to cryopreservation. In the experiment, we will simply add the suitable templates to the prefabricated 96-well PCR plate. The prefabricated 96-well PCR plates and multichannel pipettes greatly shorten the time required for desalination and purification of HLAMSTTs. The parallel experimental workflow allows the high-resolution typing to be completed within 12 hours. HLA typing using NGS generally takes 3 to 4 days and requires more complex computer hardware and analysis software (35). When comparing with NGS, the HLA MS typing has fewer experimental steps, faster processing speed, and less stringent sample quality requirements. Thus, the HLA MS typing algorithm can process experimental data in a cost-effective and timely manner. We acknowledge that, in terms of cost and time, this method does not show advantages over Sanger sequencing. The advantages of MS lie in distinguishing ambiguous typing and interpreting heterozygotes when compared with Sanger sequencing.

HLA MS typing has an advantage over other existing methods in resolving overlapping sequencing signal peaks of heterozygous samples, thus aiding the interpretation of ambiguous HLA typing results. In MS-based typing, the signals from heterozygous samples with different molecular weights are independently displayed at different positions on the spectral abscissa. In this way, the two alleles do not interfere with each other. Good signal strength and HLA name assignment for both alleles in a sample greatly improves the ability of genotyping HLA genes accurately. In Sanger sequencing, the cis/trans ambiguity of sequencing signal greatly affects the interpretation of typing results, when two different bases appear simultaneously at an SNP locus in a sample. The PCR-SSP technique also has difficulty handling the typing of heterozygous samples. MS can distinguish between two different alleles in heterozygous samples, mainly because of the following system characteristic: MS ionization and electric field make the two HLAMSTTs gather in different places because their flight times are different. TA cloning can also analyze and type the two alleles separately, which is a reliable typing method for heterozygous samples. However, TA cloning is too time consuming.

Compared with the current HLA typing methods, the MS-based method has other advantages. Unlike the SSO method, which requires high-stringency posthybridization washes and precisely controlled hybridization conditions to avoid false positive or negative results, the MS method does not rely on hybridization to distinguish between alleles (36). MS can obtain high-resolution typing results more easily than SSO or SSP. In SBT, a heterozygous peak (two peaks at the same position due to an SNP) is difficult to distinguish from the background signal, especially when the signal of one allele is too low (37, 38). The anti-interference capability and high sensitivity of MS solves this problem. The anti-interference capability of MS typing arises from the fact that only those signal peaks whose molecular weights exist in the HLA MS database are searched by the software. It eliminates interference from contaminating or background signals. The high sensitivity of MS typing also means that it is able to process samples of low quality or concentration. Signals from trace amounts of samples can be clearly read from mass spectra. In addition, MS can separate out mixed samples and determine the nature of sample components, even if the amount of one component is far lower than that of the other. The minimum molecular weight difference between the four deoxyribonucleic acids is 9 Da (T to A substitution), which means high-performance MS can easily distinguish SNPs from normal instrument error (25). Depending on the performance and calibration of the instrument, the error may be reduced even further, but it will not have any impact on the final typing result. Although the MS HLA typing method has numerous strengths, it also has some limitations. When analyzing DNA fragments longer than 150 bp, the accuracy and success rates will be reduced due to the ionization characteristics of MS, which makes it difficult for us to analyze haplotypes containing only two SNPs located over 100 bp apart (50bp reserved for primers). MS-based typing methods cannot directly define mutations. When new HLA typing occurs, the typing software may prompt HLA typing outside the database. We need to confirm it in other ways, such as NGS. Not all typing results are high-resolution. The accuracy of the MS typing method can reach 100% at the first 4 digits and 97.1% at the first 6 digits.

Considering the high accuracy of the MS method, it can not only be used as a complete method for HLA typing, but it can also replace PCR-SSP as a method for rapid heterozygosity verification. In conclusion, we used the MS and Sanger methods to type a group of HLA-DQA1 samples and obtained consistently accurate typing results. The experiment proves that the HLA MS typing algorithm software and experimental method are reliable and readily applicable to the typing of highly polymorphic gene regions. It is feasible to apply the genotyping function of this software to other gene regions by importing these gene databases and adjusting the software parameters.

## Data availability statement

The raw data supporting the conclusions of this article will be made available by the authors, without undue reservation.

## Ethics statement

The studies involving human participants were reviewed and approved by Medical Ethics Committee of Shenzhen Blood Center SZBC2019R021. Written informed consent for participation was not required for this study in accordance with the national legislation and the institutional requirements.

## Author contributions

The study was designed by FeiZ, YX, KW, FengZ. Experiments and data analysis were performed by KW and ZS with advice from FengZ and YX. The results were interpreted by KW. The paper was written by KW with advice from all the authors. All authors contributed to the article and approved the submitted version.
